# Comparison of SPME Methods for Determining Volatile Compounds in Milk, Cheese, and Whey Powder

**DOI:** 10.3390/foods2040534

**Published:** 2013-11-27

**Authors:** Michael H. Tunick, Susan K. Iandola, Diane L. Van Hekken

**Affiliations:** Dairy & Functional Foods Research Unit, Eastern Regional Research Center, Agricultural Research Service, United States Department of Agriculture, 600 E. Mermaid Lane, Wyndmoor, PA 19038, USA; E-Mails: susan.iandola@ars.usda.gov (S.K.I.); diane.vanhekken@ars.usda.gov (D.L.H.)

**Keywords:** GC-MS, milk, Queso Fresco, SPME, whey powder

## Abstract

Solid phase microextraction and gas chromatography-mass spectrometry (SPME-GC-MS) are commonly used for qualitative and quantitative analysis of volatile compounds in various dairy products, but conditions have to be adjusted to maximize release while not generating new compounds that are absent in the original sample. Queso Fresco, a fresh non-melting cheese, may be heated at 60 °C for 30 min; in contrast, compounds are produced in milk when exposed to light and elevated temperatures, so milk samples are heated as little as possible. Products such as dehydrated whey protein are more stable and can be exposed to longer periods (60 min) of warming at lower temperature (40 °C) without decomposition, allowing for capture and analysis of many minor components. The techniques for determining the volatiles in dairy products by SPME and GC-MS have to be optimized to produce reliable results with minimal modifications and analysis times.

## 1. Introduction

Bovine milk contains approximately 3.5% protein, three-fourths of which is casein; the remainder consists of whey proteins, especially β-lactoglobulin and α-lactalbumin [1]. When milk is made into cheese, the casein is coagulated and the whey is drained and saved for other uses. Frequently the whey is dried and filtered to obtain whey protein concentrate (WPC, 29%–89% protein) and whey protein isolate (at least 90% protein), which are sold as dietary supplements and food ingredients [2]. Aged cheese contains a multitude of flavor compounds from lipolysis, lipid oxidation reactions, and carbohydrate catabolism, with proteolysis providing mostly basic tastes and background flavors. Milk is a relatively bland food that is discarded when it starts to go sour, and therefore is not consumed once bacteria generate an undesirable amount of lactic acid. WPC contains little fat and is also bland.

The flavor profiles of milk, cheese, and whey protein concentrate/isolate are important factors in consumer acceptance. The compounds responsible for flavor in food are usually identified and quantitated by extracting them from the sample matrix and passing them through a gas chromatograph with a mass spectrometer detector (GC-MS). Flavor compounds in dairy products are often isolated by solid phase microextraction (SPME) [3], developed by Pawliszyn and coworkers in 1990 as a method of preparing samples for chromatographic analysis without using solvents [4,5]. In SPME, volatile compounds are adsorbed on a stationary phase that is coated on a fused silica fiber. The fiber is inserted into the headspace above the sample, which has been allowed to equilibrate at 40–60 °C for 15–30 min, and is exposed for 5–30 min. Liquid samples are frequently added to a concentrated NaCl solution to decrease solubility of the volatile compounds and force them into the headspace. The compounds adsorbed on the fiber are then thermally desorbed for 5 min in the GC injector port and then sent through the column and detector. The combination of SPME and GC-MS has been used to examine the flavor of milk [6], cheese [7], and whey solution [8].

SPME is an equilibrium extraction and desorption process, so precise control of sampling conditions is important [9]. Recoveries may be enhanced by raising the temperature and time, but compounds may be displaced from the absorption sites by doing this [9] and compounds that were not originally present in the sample may be formed. Therefore, the procedures employed must be applicable to the type of sample to prevent spurious results. This paper will show that SPME procedures for analyzing flavor compounds in milk, WPC, and Queso Fresco (QF), a non-melting cheese variety, must be varied to determine optimal conditions and meaningful results.

## 2. Experimental Section

### 2.1. Samples

Samples of homogenized and pasteurized conventional and organic milk were purchased at local markets. The WPC (Leprino Foods, Denver, CO, USA) was labeled as containing 81.5% protein. The QF was prepared in the laboratory’s pilot plant using the procedure of Guo *et al.* [10] and was stored at 4 °C prior to sampling.

### 2.2. Solid-Phase Microextraction

The milk and QF samples were held at −80 °C until analysis. To avoid contamination by volatiles in the laboratory, the samples were defrosted at 24 °C in a refrigerator used only for that purpose. The WPC was stored at 20 °C. Samples, consisting of 10 mL milk or 0.2 g WPC dissolved in 10 mL water or 5 g finely diced QF, were placed in a 20-mL amber glass vial with a poly(tetrafluoroethylene)/silicone septum (Supelco, Bellefonte, PA, USA) along with 10 μL of 100 ppm 2-methyl-3-heptanone, which served as the internal standard (Sigma Aldrich, St. Louis, MO, USA). Four grams NaCl (Sigma Aldrich) was added to the liquid samples followed by 10 s of agitation at 500 rpm. The sample was equilibrated at 21 or 40 °C for various lengths of time. A 2-cm, 50/30-μm film thickness DVB/Carboxen/PDMS Stableflex SPME fiber (Supelco) was exposed to the sample with continuous stirring at 40 °C for 30–60 min. The analytes were desorbed for 5 min from the fiber onto the column through a splitless injector at 250 °C. A Varian gas chromatograph (model CP 3380, Varian, Walnut Creek, CA, USA) equipped with a 30-m, 0.25-mm i.d., 0.25-μm film thickness DB-5 column (Restek US, Bellefonte, PA, USA) and a Varian Saturn 2000 mass spectrometer were used to separate and identify volatile compounds. The flow rates of the helium carrier gas and the oven temperature programming are shown in Table 1. The peak areas of volatile compounds were taken to be their relative abundances. The internal standard exhibited a peak area around 1000, which was equivalent to a concentration of 0.10 ppm.

**Table 1:** Carrier gas and oven temperature programming for milk, Queso Fresco cheese, and whey protein concentrate (WPC).

Sample	Flow rate (mL/min)	Initial oven temperature (°C)	Hold time (min)	Ramp speed (°C/min)	Final oven temperature (°C)	Hold time (min)
Milk	0.6	40	2	10	200	5
Cheese	1.0	40	10	5	225	5
WPC	1.0	40	3	10	250	5

## 3. Results and Discussion

### 3.1. Milk

Organic and conventional milk samples were analyzed using different equilibrating and sampling conditions (Table 2). All samples underwent an equilibration step at 40 °C for 1, 5, or 30 min; one sample (Experiment 4) was held at 21 °C for 90 min prior to this step and another (Experiment 2) was held at 21 °C for 60 min following this step. The volatiles were adsorbed onto the SPME fiber at 40 °C for 30 or 60 min, and some samples (Experiments 6 and 7) were equilibrated at 21 °C for various times before injection.

A comparison of the results is shown in Table 2. The total relative abundances increased with time and temperature. Almost all of the volatile organic milk compounds exhibited a higher relative abundance than the conventional milk compounds, indicating that the organic milk, which came from cows eating pasture plants, might be more flavorful. The relative abundance of hexanoic, octanoic, nonanoic, and decanoic acids increased with time at 40 °C and were highest when the 90-min equilibration at 21 °C was added. Fatty acids in milk arise from incomplete triacylglycerol synthesis in the cow, microbial action, and lipase activity [11]. Indigenous lipases and bacteria apparently cleaved triacylglycerides during the period before injection. The methyl ketones 2-heptanone and 2-nonanone and the aldehydes nonanal, pentanal (in the organic milk only), and 2-methylbutanal and heptanal (in the conventional milk only) tended to increase with equilibration time, often from undetectable levels. Aldehydes and ketones are lipid oxidation products from breakdown of fatty acids [12]. Methyl ketones with 7–11 carbon atoms have been found by others to increase in concentration in milk with heating intensity [13]. Methyl propionate (in the conventional milk only) and 1-pentanol (in the organic milk only) may have arisen from microbial activity [14]. Dimethyl sulfide and dimethyl sulfone, from thermal decomposition of cysteine and methionine [14], were found in the organic milk samples that were treated for >1 h. α-Pinene, present only in the organic milk, is a terpenoid found in pasture plants [15]. Small peaks corresponding to naphthalene, its saturated analog decalin, and several other naphthalene derivatives were seen in the organic milk from 10.2 to 12.2 min (these numbers were not included). The presence of polycyclic aromatic hydrocarbons in milk has been attributed to pollution from sources not necessarily close to the farm [16].

**Table 2:** Relative abundance of volatile compounds in milk, and variations with temperature conditions.

Experiment		Organic milk				Conventional milk		
		1	2	3	4	5	6	7
*Temperature steps*		*Minutes per step*						
Equilibration at 21 °C		0	0	0	90	0	0	0
Equilibration at 40 °C		1	1	30	30	5	5	5
Holding at 21 °C		0	60	0	0	0	0	0
Adsorption at 40 °C		60	60	60	60	60	30	60
Holding at 21 °C		0	0	0	0	0	30	60
*Compound*	*Retention time (min)*	*Relative abundance (thousands)*						
2-Methylbutanal	3.4	0	0	0	0	0	19	27
Pentanal	3.8	26	46	29	122	0	0	0
Dimethyl sulfide	4.5	0	9	0	22	0	0	0
Methyl propionate	4.6	0	0	0	0	45	111	155
1-Pentanol	4.9	0	0	14	33	0	0	32
Hexanal	5.5	0	0	0	22	10	17	41
Butanoic acid	5.6	0	18	14	22	0	0	0
2-Heptanone	7.1	78	64	87	156	4	11	18
Heptanal	7.3	0	0	0	0	0	0	23
Dimethyl sulfone	7.7	13	18	7	11	0	0	0
α-Pinene	8.0	39	32	29	33	0	0	0
Hexanoic acid	8.6	317	59	108	133	0	0	0
2-Nonanone	10.6	100	91	123	155	0	0	0
Nonanal	10.8	0	0	87	56	7	13	18
Octanoic acid	11.8	74	68	159	244	12	28	82
Nonanoic acid	13.2	9	5	14	33	0	0	0
Decanoic acid	14.6	48	32	36	89	0	11	50
2-Methylpropanoic acid	14.8	22	9	7	0	4	4	14

The number of compounds detected in these milk samples was less than the number found by others in raw milk. For instance, Croissant *et al.* [14] identified 33 compounds in raw milk from conventionally- and pasture-fed cows. Pasteurization inactivates nearly all indigenous enzymes and microbes in milk, resulting in greatly reduced compound formation between milking and analysis. Contarini and Povolo examined sterilized milk samples subjected to pasteurization above 120 °C and found fewer than 12 compounds [17].

In a separate experiment, samples from one carton of conventional whole milk were prepared at the same time and held in the autosampler at room temperature before they were sequentially exposed to the SPME fiber and injected. The relative abundance for each of the major compounds increased from 2.50 to 5.67 h and, with the exception of the aldehydes, remained steady or slightly decreased afterwards (Table 3). The initial increases, and the continued increases by octanal and nonanal, were apparently due to compound formation. The later decreases may have been caused by breakdown into other products.

The results indicate that extending the analysis time of milk samples will increase the number of compounds detected. Some relative abundance numbers were elevated due to enhanced compound formation whereas other numbers fell as further reactions occurred. Moreover, extracting at a high temperature may concentrate higher molecular weight compounds on the fibers and displace lower molecular weight volatiles due to an SPME film capacity effect [18]. It must be emphasized that if the analysis of the compounds present in unheated refrigerated milk is the goal, its analysis times should be kept to a minimum.

**Table 3:** Relative abundance of volatile compounds in milk and variations with equilibration time.

Compound	Retention time (min)	Time at 21 °C (h)			
		2.50	5.67	7.33	9.00
		Relative abundance (thousands)			
1-Pentanol	4.9	22	40	40	40
Butanoic acid	5.1	30	50	40	40
Dimethyl sulfone	7.5	15	20	20	20
Hexanoic acid	8.8	400	450	450	450
Octanal	9.1	18	20	30	35
Nonanal	10.8	25	25	120	140
Octanoic acid	11.9	400	560	550	550
Nonanoic acid	13.2	22	60	65	60
Decanoic acid	14.6	120	300	310	280

### 3.2. Cheese

Higher analysis temperatures may be used for QF since this variety does not melt. Equilibrating and extracting at 60 °C produced better resolution of peaks than at 50 °C, so a holding period of 60 °C for 10 min was used. When samples of a QF frozen for 42 weeks were extracted onto the fiber at 60 °C for 60 min, the relative abundance of most of the volatile compounds was lower than when the extraction lasted 30 min (Table 4). The longer time may have caused the fiber to become saturated and lose volatiles, so the shorter time was optimal.

Table 5 shows the volatile compounds in the QF cheeses stored for up to 8 weeks. Because of variations between the many cheeses analyzed, categories of relative abundance are given instead of averages. Many of the compounds present in the milk samples were also detected in the cheese. The acids and ketones increased with storage as did nonanal and decanal, which continued to form from lipolysis of longer-chain fatty acids. In contrast, the other aldehydes decreased to undetectable levels. The shorter-chain aldehydes are relatively transient compounds in cheese, breaking down into alcohols and acids [19]. Aldehydes were absent from the 42-week cheese, and five different alcohols were present. Nonalactone was not observed in the milk but is a common volatile compound in cheese. Furaneol and esters were first detected after 4 weeks storage. Furans such as furaneol, which has an IUPAC nomenclature of 4-hydroxy-2,5-dimethyl-3-furanone, are formed in dairy products by some Lactobacilli strains [20].

**Table 4:** Relative abundance of volatile compounds in Queso Fresco when extracted at 60 °C for 30 or 60 min.

Compound	Retention time (min)	Extraction time (min)	
		30	60
		Relative abundance (thousands)	
2-Pentanone	3.1	920	620
2-Pentanol	3.3	1400	700
3-Methyl-1-butanol	4.1	500	260
2-Hexanone	5.7	100	100
Ethyl butanoate	6.3	420	200
1-Hexanol	10.7	150	175
2-Heptanone	12.2	3200	3200
2-Heptanol	13.0	2400	1900
1-Heptanol	16.5	40	40
Octanoic acid	24.8	500	360
Ethyl octanoate	25.0	375	200
Furanone	26.7	55	0
Nonanoic acid	27.1	44	0
2-Undecanone	27.8	1160	1120

**Table 5:** Relative abundance of volatile compounds in Queso Fresco, arranged by compound class, and their variation with storage time at 4 °C. ND = not detected, L = 0–9000, M = 10,000–49,000, H = 50,000–99,000, VH = above 100,000.

	Storage time (week)		
	1	4	8
*Acids*			
Acetic	ND	M	M
Butanoic	ND	ND	M
Octanoic	M	M	VH
Nonanoic	ND	ND	M
Decanoic	M	M	H
Dodecanoic	M	L	M
*Aldehydes*			
Pentanal	M	M	ND
Hexanal	M	M	ND
Heptanal	L	L	ND
Nonanal	M	VH	VH
Decanal	L	L	M
*Ketones*			
2-Heptanone	L	L	M
2-Nonanone	L	L	M
Nonalactone	L	M	H
*Others*			
Furaneol	ND	L	M
Methyl propionate	ND	ND	M
Ethyl decanoate	ND	M	VH

As with milk, the number and relative abundance of compounds that are detected in cheese by SPME-GC-MS increases with analysis time. Frank *et al.* [21] found 45 volatile compounds in commercial Cheddar cheeses that were exposed to a SPME fiber for 16 h at 22 °C whereas Chin *et al.* [7] identified just 15 compounds in Cheddar, Romano, and Swiss cheeses that were exposed for 20 min at 60 °C. Burbank and Qian [22] exposed Cheddar cheese to a SPME fiber for up to 120 min at 50 °C and found logarithmic or power law relationships between sulfur compound levels and time. They found unpredictable trends when the time was held constant at 30 min and the temperature was varied from 30 to 70 °C.

QF is made without starter culture bacteria, so it contains fewer flavor compounds than most varieties. Far more volatiles are present in odiferous cheeses; Pérès *et al.* [23] found over 70 compounds in Camembert after 10 min of extraction at room temperature. Therefore, analysts should account for the number of volatiles likely to be in a sample and experiment with time and temperature conditions.

### 3.3. WPC

Quach *et al.* [8] studied conditions for SPME-GC-MS of WPC and found that extracting the sample at 40 °C for 30 min produced better results than holding for 1 h at 40 °C or for 2 h at 27 or 50 °C. They also found that a 30-min equilibration period was necessary to allow the less volatile compounds to be liberated from the sample. The same conditions we used for QF—holding at 60 °C for 10 min, extracting at that temperature for 30 min, and ramping the oven temperature at 5 °C/min—were compared with a procedure developed by Wright *et al.* [24], in which the sample was equilibrated at 40 °C for 30 min and extracted at 40 °C for 30 min, and the oven was ramped at 10 °C/min.

The results using the two methods are shown in Table 6. Unlike milk and cheese, where the principal proteins are α_s1_- and β-casein, almost all of the proteins in whey consist of β-lactoglobulin, α-lactalbumin, and bovine serum albumin. The differences in structure (e.g., whey proteins contain cysteine and α_s1_- and β-casein do not [25]) result in different volatile compounds being formed. The faster ramping in the Wright method [24] allowed the analysis to be completed much earlier than the QF method, the total relative abundance was higher, and eight compounds not detected using the other method were observed. However, six 8-, 9- and 10-carbon compounds not observed with the Wright method [24] were found toward the end of the analysis using the slower QF method. The amounts of hexanal and nonanal in the two methods differed substantially since nonanal is one of the compounds eluted late with the Wright method [24] and exhibited a low relative abundance. These aldehydes are important since they affect whey protein aroma and were found by Carunchia Whetstine *et al.* [26] to have the highest relative abundance among non-acidic volatiles in WPC.

**Table 6:** Relative abundance of volatile compounds in whey protein concentrate, and variations with temperature conditions. ND = not detected.

Compound	60 °C hold for 10 min		40 °C hold for 25 min	
	Retention time (min)	Relative abundance (thousands)	Retention time (min)	Relative abundance (thousands)
3-Methylbutanal	2.7	2600	2.7	69,800
2-Methylbutanal	3.3	11,300	2.8	80,100
3,3-Dimethyl-2-butanone	ND		3.5	49,100
Dimethylsulfide	ND		4.1	20,700
1-Pentanol	5.1	26,000	ND	
3-Hexanone	5.6	57,900	4.9	36,200
2-Hexanone	ND		5.0	18,100
Hexanal	6.3	422,300	5.3	1,654,100
3-Ethyl-2-pentanone	ND		4.3	28,400
3-Heptanone	ND		7.0	20,700
2-Heptanone	ND		7.1	43,900
1-Heptanal	12.9	59,300	7.4	124,100
2-Heptanal	ND		8.5	12,900
Heptanol	16.6	7200	8.7	15,500
2-Pentylfuran	ND		9.1	49,100
1-Octen-3-ol	17.0	28,900	ND	
2-Ethylhexanol	19.1	59,300	9.8	124,100
Nonanol	20.8	23,100	ND	
2-Nonen-1-ol	21.3	5800	9.9	23,300
Nonanal	22.0	458,500	11.1	121,500
Octanoic acid	24.2	65,100	ND	
Decanal	25.3	18,800	ND	
Nonanoic acid	27.1	36,200	ND	
Decanoic acid	29.8	89,700	ND	

It appears that the best compromise between the two methods is simply to use both for each sample. By doing this, the entire range of volatiles can be observed.

## 4. Conclusions

In summary, when investigating dairy products by SPME-GC-MS, a balance must be struck between detecting the compounds that are present, avoiding the formation of compounds that are absent in the sample, and completing the analysis in a timely manner.
